# Association of Circulating IgE and CML Levels with In-Stent Restenosis in Type 2 Diabetic Patients with Stable Coronary Artery Disease

**DOI:** 10.3390/jcdd9050157

**Published:** 2022-05-13

**Authors:** Jingmeng Liu, Qiujing Chen, Lin Lu, Qi Jin, Yangyang Bao, Tianyou Ling, Changjian Lin, Fenghua Ding, Xiaoqun Wang, Weifeng Shen, Ying Shen, Yang Dai, Liqun Wu

**Affiliations:** 1 Department of Cardiovascular Medicine, Ruijin Hospital, Shanghai Jiao Tong University School of Medicine, Shanghai 200025, China; liujingmeng@163.com (J.L.); cqj40575@rjh.com.cn (Q.C.); rjlulin1965@163.com (L.L.); jinqi127@163.com (Q.J.); oliverbao@icloud.com (Y.B.); lty0919@hotmail.com (T.L.); linchangjian222@126.com (C.L.); ruijindfh@126.com (F.D.); xiaoqun_wang@hotmail.com (X.W.); rjshenweifeng@126.com (W.S.); 2 Institute of Cardiovascular Diseases, Ruijin Hospital, Shanghai Jiao Tong University School of Medicine, Shanghai 200025, China

**Keywords:** immunoglobulin E, Nε-carboxymethyl-lysine, in-stent restenosis, diabetes, percutaneous coronary intervention

## Abstract

Background: We investigated whether serum levels of immunoglobin (Ig) E and Nε-carboxymethyl-lysine (CML) are related to in-stent restenosis (ISR) in patients with stable coronary artery disease and type 2 diabetes mellitus (T2DM). Methods: Serum levels of IgE and CML were measured in 196 ISR patients and 220 non-ISR patients with stable angina and T2DM who received angiographic follow-up 12 months after percutaneous coronary intervention (PCI) with third-generation drug-eluting stent (DES) implantation for de novo lesions. Multivariate logistic regression analysis was performed to assess the association between IgE or CML and ISR. Results: Both IgE and CML levels were higher in patients with ISR compared with non-ISR patients (IgE: 187.10 (63.75–489.65) vs. 80.25 (30.65–202.50), *p* < 0.001; CML: 203.26 (164.50–266.84) vs. 174.26 (130.85–215.56), *p* < 0.001). The rate of ISR increased stepwise with increasing tertiles of IgE and CML levels (*p* for all trends < 0.001), and IgE correlated significantly with CML. After adjusting for potential confounders, IgE and CML levels remained independently associated with ISR. Moreover, IgE and CML levels improved the predictive capability of traditional risk factors for ISR, and there existed an interaction between IgE and CML in relation to ISR (*p* for interaction < 0.01). Conclusion: Elevated circulating IgE and CML levels confer an increased risk for ISR after DES-based PCI in type 2 diabetic patients with stable coronary artery disease.

## 1. Introduction

Despite the widespread use of drug-eluting stents (DES), in-stent restenosis (ISR) remains a significant clinical problem after percutaneous coronary intervention (PCI) [1], occurring in 3% to 20% of patients after DES implantation [2,3]. The prevalence of ISR is even higher in patients with type 2 diabetes mellitus (T2DM), which causes particular concern since the diabetic population is growing and these patients often have more severe and diffuse coronary artery disease, requiring DES-based revascularization [4]. The mechanism of ISR in type 2 diabetic patients remains incompletely elucidated but is likely to be multifactorial. Apart from mechanical and technical factors, as well as patient- and operator-related conditions, certain biochemical abnormalities and inflammatory cytokines induced by diabetes have been documented to exaggerate neointimal hyperplasia and to promote ISR [1,5,6].

Advanced glycation end products (AGEs), which form more abundantly during T2DM as a consequence of chronic hyperglycemia, are extensively distributed in the diabetic vasculature. Mounting evidence has indicated that AGEs play a key role in cell signaling to accelerate vasculopathy in diabetes. For example, they react with receptors for AGEs (RAGE) to increase oxidative stress, the expression of transforming factor-β, and extracellular matrix accumulation [7]. Elevated AGEs have been suggested as a risk factor for post-PCI restenosis as well as coronary artery disease progression in type 2 diabetic patients [8,9]. Nε-carboxymethyl-lysine (CML), a major isoform of AGE, contributes to endothelial dysfunction in diabetes and is associated with cardiovascular mortality [10,11,12]. Nevertheless, the relationship between CML and ISR remains unclear.

Immunoglobin (Ig) E, despite its low abundance in vivo, exerts a crucial effect in mediating type I hypersensitivity both systematically and locally, and defending against pathogens as the first line of defense [13]. Previous studies have demonstrated that elevated IgE levels are most common in allergies, and interestingly, the risk of cardiovascular diseases such as acute myocardial infarction, heart failure, atrial fibrillation, and peripheral vascular disease is increased in patients with allergic disorders [14,15,16,17]. Recent data revealed that IgE promotes coronary atherosclerosis [18] and participates in abdominal aortic aneurysm formation [19] and coronary artery spasm, independent of atheromatous disease [20]. Likewise, elevated IgE levels in serum have been shown to correlate with multivessel disease and contribute to discriminating coronary artery disease severity [21].

Although the formation of AGEs has been reported to correlate with immunological and allergic disorders such as asthma and arthritis [22], the exact role of allergic inflammation in both the pathogenesis of coronary artery disease and the occurrence of adverse events following stent implantation has just begun to be noticed in recent years [23]. In addition, knowledge regarding the interactions between IgE and CML on ISR is still scarce. In this study, we sought to examine if circulating levels of IgE and CML are associated with ISR in patients with T2DM after PCI with DES implantation. 

## 2. Materials and Methods

This study was approved by the ethics committee of Ruijin Hospital, Shanghai Jiao Tong University School of Medicine, and conducted according to the Declaration of Helsinki. Written informed consent was obtained from all participants.

### 2.1. Clinical Cohort

**Figure 1:**
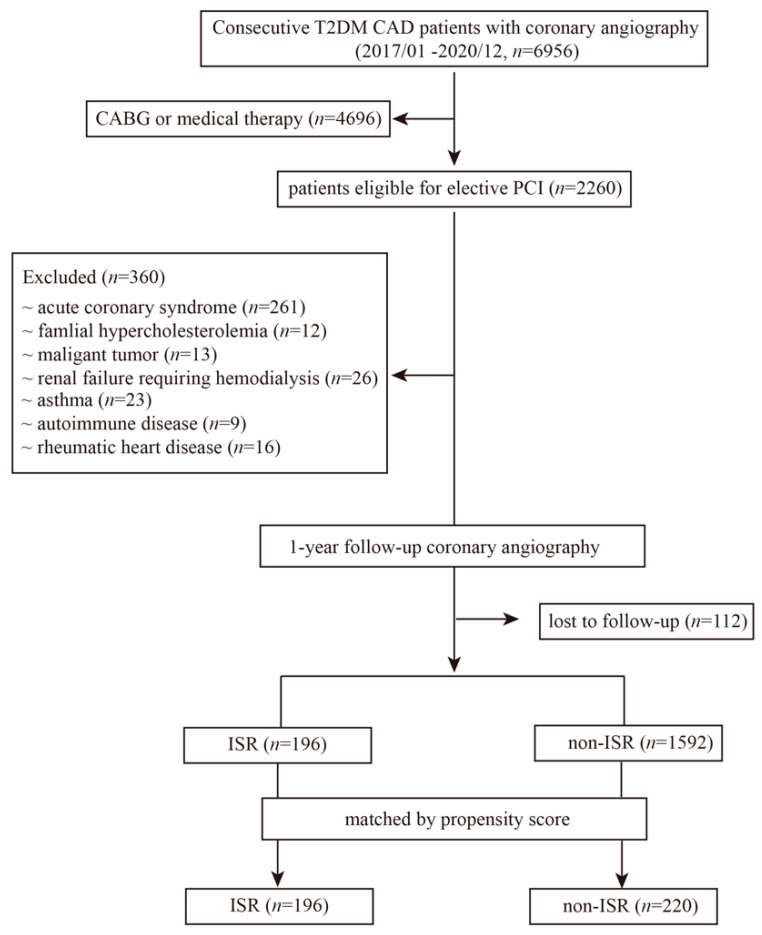
Flow chart of recruitment procedure. T2DM: type 2 diabetes mellitus; CAD: coronary artery disease; PCI: percutaneous coronary intervention; CABG: coronary artery bypass grafting; ISR: in-stent restenosis.

In 6956 consecutive patients with T2DM who received coronary angiography from January 2017 to December 2020 in Shanghai Ruijin Hospital, 2260 patients were eligible for elective PCI. For the purposes of this study and to avoid confounding serum data, we excluded patients with acute coronary syndrome (*n* = 261), familial hypercholesterolemia (*n* = 12), malignant tumors (*n* = 13), and renal failure requiring hemodialysis (*n* = 26). Patients with a history of asthma (*n* = 23), autoimmune disease (*n* = 9), or rheumatic heart disease (*n* = 16) were also excluded. A total of 112 patients were lost to 1-year follow up. Then, after being matched by propensity score (including age, sex, BMI, smoking, hypertension, dyslipidemia, serum creatinine, and eGFR), 196 ISR patients and 220 non-ISR patients were eligible and categorized in the final analysis for IgE and CML at 1-year follow-up (Figure 1). 

Diagnoses of T2DM were made according to the criteria of the American Diabetes Association (symptoms of diabetes with casual plasma glucose concentration ≥ 200 mg/dL (11.1 mmol/L) or fasting plasma glucose ≥ 126 mg/dL (7.0 mmol/L), 2 h postprandial glucose ≥ 200 mg/dL (11.1 mmol/L) during an oral glucose tolerance test, and currently or previously treated with insulin and/or oral hypoglycemic agents) [24]. Hypertension was diagnosed according to the seventh report of the Joint National Committee on the prevention, detection, evaluation, and treatment of high blood pressure (JNC 7) [25]. 

### 2.2. Coronary Angiography and Quantitative Analysis

Coronary angiography and PCI were performed through the radial or femoral approach using standard methods. All lesions were stented with a normal-to-normal technique, usually including 5 mm-long, angiographically normal segments proximal and distal to the lesion. The third-generation DES was applied to all patients, but the choice of stent type and technique of deployment were left to the discretion of the operators. A plurality of matching angiographic images was obtained after intracoronary nitrate injection for each patient. All patients were encouraged to take guideline-recommended medications after the procedure.

End-diastolic frames from both baseline and follow-up angiograms were selected with identical angulations that best showed the stenosis at its most severe degree, with minimal foreshortening and branch overlap [26]. Quantitative coronary analysis (QCA) of baseline and follow-up angiograms was performed using Cardiovascular Measurement System version 3.0 software (Terra, GE, USA) by two experienced cardiologists who were blinded to patients’ clinical information and biochemical measurements. Briefly, the outer diameter of a contrast-filled catheter was used for calibration to determine absolute measurements in millimeters. Lesion length was measured as the distance from the proximal to the distal shoulder. A value of 0 mm was assigned as the minimal lumen diameter in cases of total occlusion at baseline. ISR was defined as recurrence of a luminal diameter stenosis of >50% within the stent or in the 5 mm proximal or distal segments adjacent to the stent at follow-up angiography [26]. For patients with multiple coronary lesions, the most severe restenotic lesion at follow-up was included in the analysis.

### 2.3. Biochemical Assessments

Peripheral venous blood samples were obtained at the day of angiography after overnight fasting. To avoid a diurnal variation in IgE concentration and dramatic fasting interval effects, all blood samples were obtained at 8:00 a.m. Serum levels of glucose, blood urea nitrogen, uric acid, and creatinine, and lipid profiles including triglyceride, total cholesterol, low-density lipoprotein (LDL) cholesterol, high-density lipoprotein (HDL) cholesterol, lipoprotein (a), apolipoprotein A-I, and apolipoprotein B were measured using standard laboratory techniques on a HITACHI 912 Analyzer (Roche Diagnostics, Germany). Blood concentrations of glycosylated hemoglobin (HbA1c) were measured using ion-exchange high performance liquid chromatography with a Bio-rad Variant Hemoglobin Testing System (Bio-Rad Laboratories, Hercules, CA, USA). Serum levels of high-sensitivity C-reactive protein (hsCRP) were determined by ELISA (Biocheck Laboratories, Toledo, OH, USA). The estimated glomerular filtration rate (eGFR) was calculated using the Chronic Kidney Disease Epidemiology Collaboration equation [27]. 

Serum levels of IgE and CML were determined by enzyme-linked immunosorbent assay (ELISA) according to the manufacturer’s protocols (BMS2097, eBioscience, Carlasbad, CA, USA; STA-816, Cell BioLabs, San Diego, CA, USA). The average inter-assay coefficient of variance (CV) was 6.2% and 5.8% for IgE and CML, respectively, and the average intra-assay CV was 6.6% or 7.2% for IgE or CML, respectively.

### 2.4. Statistical Analysis

All statistical analyses were performed with SPSS 26.0 (IBM, Armonk, New York, NY, USA) and R Programming Language 4.0.2. Continuous variables were expressed as mean ± standard deviation (SD) if data were normally distributed, or as median (25th–75th percentile) otherwise, and categorical variables were summarized as frequencies (percentages). IgE and CML levels were presented both as an original skewed-distributed variable and a log2-transformed normally distributed variable. Continuous variables were compared between two groups using student’s t-tests or Mann–Whitney U tests. For categorical variables, differences between groups were evaluated with the chi-square test. Pearson’s and Spearman’s correlation tests were used to assess the relationship between IgE and CML. Logistic regression models were applied to detect the relationship between ISR and serum IgE or CML levels. IgE or CML were analyzed as continuous variables with log transformation, as an ordinal variable, and as a categorical variable divided into tertiles. Odds ratios (OR) were calculated with unadjusted values, adjusted for age, sex, body mass index, smoking, dyslipidemia, and hypertension (model 1), and further adjusted by adding HbA1c, left ventricular ejection fraction, use of statins, number of diseased vessels, B2/C lesion, bifurcation, chronic total occlusion, and stent diameter (model 2). Receiver-operating characteristic (ROC) curves were plotted to determine the power of IgE and CML for detecting ISR, and the C statistics was compared using the Delong method. Category-free net reclassification improvement (NRI) and integrated discrimination improvement (IDI) were calculated to assess the added value in the reclassification of the patients. A 2-sided *p* value < 0.05 was considered statistically significant.

## 3. Results

### 3.1. Baseline Clinical Characteristics

In this clinical cohort, ISR and non-ISR were detected in 196 and 220 patients, respectively. Patients with or without ISR did not differ with respect to age, gender distribution, body mass index, risk factors for coronary artery disease, renal function, the type of DES, or follow-up duration. Blood concentrations of HbA1c and hsCRP were higher whereas left ventricular ejection fraction and statin use were lower in ISR patients. Despite a similar degree of coronary stenosis intervention and average number and length of stents implanted, patients with ISR received smaller stents and had higher percentages of circumflex or right coronary artery lesions, multivessel disease, and complex lesion morphology (Table 1).

### 3.2. IgE and CML Levels with ISR

**Figure 2:**
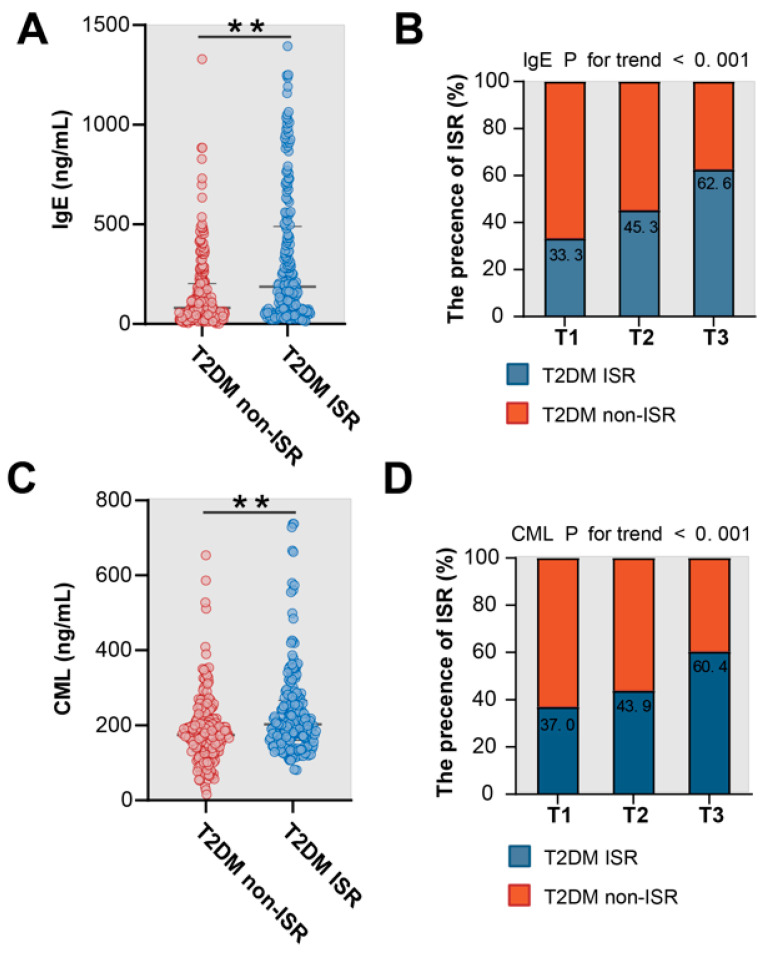
Association of serum IgE and CML levels with ISR in T2DM patients. Comparison of serum IgE (**A**) and CML (**C**) levels between patients with and without ISR in T2DM patients. In-stent restenosis across the tertiles of IgE (**B**) and CML (**D**). ISR: in-stent restenosis; T2DM: type 2 diabetes mellitus. ** *p* < 0.01. red dots: individuals with T2DM non-ISR, blue dots: individuals with T2DM ISR.

**Figure 3:**
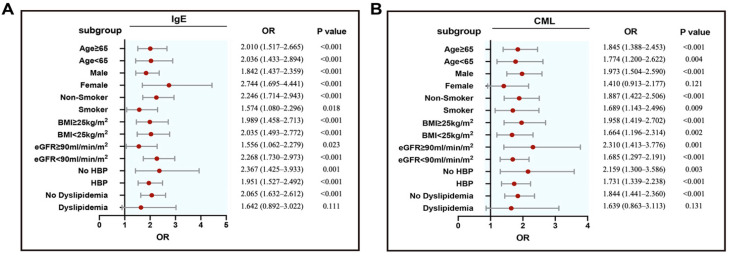
Forest plots (unadjusted) to analyze the predictive value of IgE (**A**) or CML (**B**) for ISR in different subgroups of T2DM patients. IgE or CML was included as a log- transformed continuous variable. BMI: body mass index; eGFR: estimated glomerular filtration rate; HBP: high blood pressure; ISR: in-stent restenosis; T2DM: type 2 diabetes mellitus.

Serum levels of IgE and CML were higher in ISR group compared with non-ISR group (IgE: 187.10 (63.75–489.65) vs. 80.25 (30.65–202.50), *p* < 0.001; CML: 203.26 (164.50–266.84) vs. 174.26 (130.85–215.56), *p* < 0.001). There was a stepwise increase in the incidence of ISR from the lowest tertile to the highest tertile of IgE or CML (*p* for all trends <0.001; Figure 2). Serum IgE correlated positively with CML levels (all patients: r = 0.331, *p* < 0.001; ISR group: r = 0.433, *p* < 0.001; non-ISR group: r = 0.153, *p* = 0.023), even after adjusting for confounding factors (Table 2). Logistic regression models were constructed to confirm the association between ISR and IgE or CML levels in various subgroups (Figure 3). In the multivariable analysis, both serum IgE and CML levels remained independent factors for ISR in patients with T2DM after adjusting for age, sex, body mass index, smoking, dyslipidemia, hypertension, HbA1c, left ventricular ejection fraction, use of statins, number of diseased vessel, class B2/C lesions, bifurcation, chronic total occlusions, and stent diameter. The result patterns were similar when serum IgE or CML levels were used as standardized continuous variables with log transformation and as ordinal or categorical variables (Table 3).

**Figure 4:**
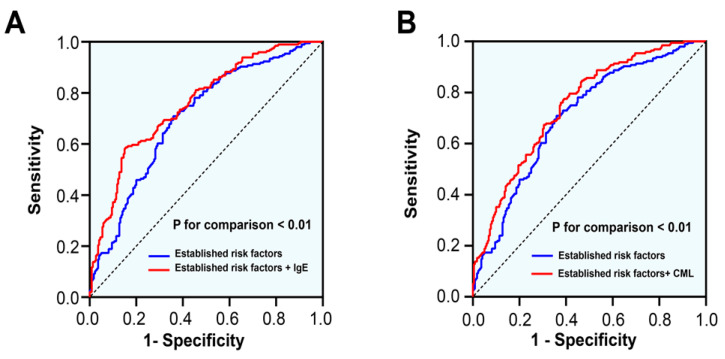
Receiver operating characteristic curve analysis between models to verify the predictive values of IgE (**A**) and CML (**B**). Established risk factors include age, sex, body mass index, smoking, dyslipidemia, hypertension, HbA1c, LVEF, statins use, number of diseased vessels, class B2/C lesions, bifurcation lesions, CTO lesions, and stent diameter.

**Figure 5:**
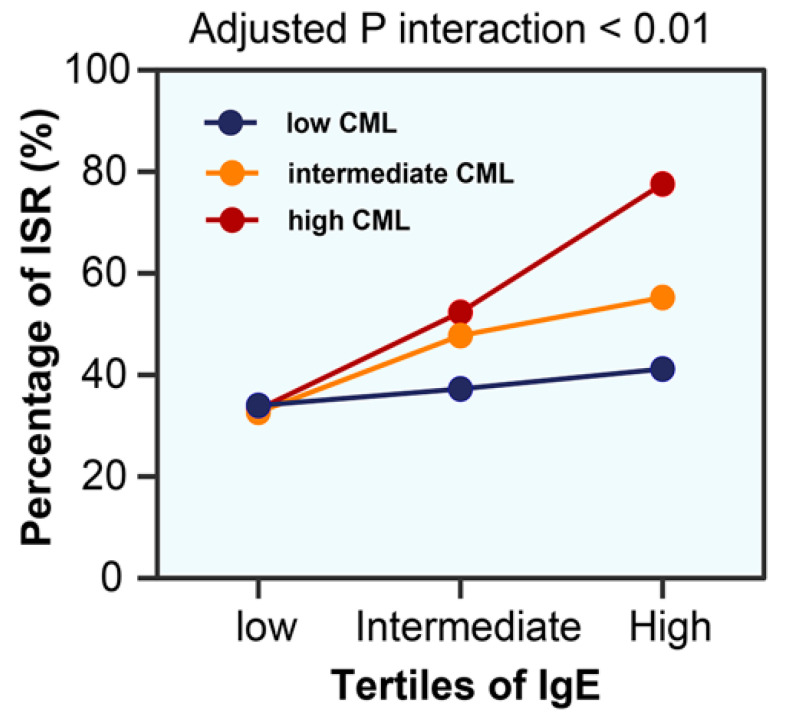
Percentage of ISR in relation to interactions between IgE and CML. ISR: in-stent restenosis.

ROC curves showed that the addition of IgE or CML to the basic clinical model significantly improved diagnostic performance for ISR in patients with T2DM (area under the curve (AUC): 0.759 (0.713–0.804) or 0.748 (0.701–0.794) vs. 0.705 (0.655–0.755), all *p* < 0.01; Figure 4). Likewise, compared to the basic clinical model, the inclusion IgE or CML showed significant improvements in reclassification as assessed by categorical NRI (0.120 (0.047–0.194); 0.099 (0.034–0.163), respectively) and IDI (0.074 (0.049–0.010); 0.062 (0.038–0.086), respectively; Table 4). More importantly, there was a significant interaction between IgE and CML in relation to ISR (*p* for interaction < 0.01). At the high tertile of IgE (≥210.7 ng/mL), patients with a high tertile of CML (≥215.5 ng/mL) had a significantly increased risk of ISR compared with those with a low tertile of CML (≤161.5 ng/mL; adjusted OR = 6.784, 95% CI 2.304–19.969, *p* = 0.001; Figure 5).

IgE and CML were analyzed as log-transformed continuous variables, ordinal variables divided according to tertiles of IgE or CML, and as categorical variables using the lowest tertile as reference. Model 1: adjusted for age, sex, body mass index, smoking, dyslipidemia, and hypertension. Model 2: adjusted for age, sex, body mass index, smoking, dyslipidemia, and hypertension, HbA1c, LVEF, statin use, number of diseased vessels, class B2/C lesions, bifurcation lesions, CTO lesions, and stent diameter. As a continuous variable, OR is shown as per one SD (standard deviation). OR: odds ratio; HbA1c: glycated hemoglobin A1c; LVEF: left ventricular ejection fraction; CTO: chronic total occlusion.

Established risk factors included age, sex, body mass index, smoking, dyslipidemia, hypertension, HbA1c, LVEF, statin use, number of diseased vessels, class B2/C lesions, bifurcation lesions, CTO lesions, and stent diameter. ISR: in-stent restenosis; NRI: net reclassification improvement; IDI: integrated discrimination improvement

## 4. Discussion

This study is the first to show that in type 2 diabetic patients with stable coronary artery disease, IgE correlates positively with CML. Elevated IgE and CML were associated with ISR after DES-based PCI, independent of traditional risk factors. 

### 4.1. Role of Elevated Circulating IgE and CML in ISR

In the bare metal stent era, the stainless-steel struts may act as a foreign body to induce effector cells of hypersensitivity, increasing IgE release and participating in ISR [28,29]. Our study cohort is unique as all patients had stable angina and received third-generation DES, which reflects current clinical practice well. The technology for new DES has been improved dramatically, often consisting of a cobalt–chromium alloy platform, an antiproliferative drug (such as everolimus or zotarolimus), and a biodegradable polymer-coating with enhanced biocompatibility [30]. Nevertheless, the stent being an exogenous substance may cause several reactions by promoting the proliferation of vascular smooth muscle cells, immune responses, and neointima hyperplasia after implantation, leading to ISR and late thrombosis. 

The major finding of this study is that serum IgE levels were higher in patients with ISR compared to the non-ISR group. Furthermore, the incidence of ISR increased stepwise across the tertiles of serum IgE and circulating IgE levels remained an independent factor for ISR in patients with T2DM after adjusting for potential confounders. These observations jointly support the notion that allergic inflammation to stents contributes, at least partly, to the development of ISR in type 2 diabetic patients after DES-based PCI. Finn et al. reported that allergy-mediated inflammation plays a more critical role in DES-related ISR than that with bare metal stent–related ISR [31]. Previous studies have shown that IgE is associated with diabetes status and may be an independent risk factor for pre-diabetes and diabetes [32,33]. IgE induces platelet activation and aggregation [34] and arterial smooth muscle hyperplasia [35], which are essential in the pathophysiology of ISR [36]. More importantly, IgE activates mast cells and basophils by binding to the high-affinity receptor FcεRI [37], and induces the release of preformed inflammatory mediators and the de novo synthesis and secretion of cytokines, chemokines, and eicosanoids, which may cause adverse events after stent implantation [23,38]. Based on these findings and our results, we speculate that in diabetic patients who have elevated serum IgE, vascular injury resulting from balloon dilatation and stent implantation could increase further IgE levels and activate allergic inflammation and relevant effector cells, potentially facilitating the occurrence of ISR. 

Another finding of this study is that CML, one of the most typical AGEs that have been implicated in diabetes-related complications [8,10], was correlated with ISR in patients with T2DM. Abundant evidence has demonstrated that the production of AGEs is not only a sign of high blood sugar, but also reflects cumulative metabolic burden, oxidative stress, and inflammation [39]. AGEs elicit the secretion of inflammatory cytokines in basophils, which are thought to play a pivotal role in allergic reactions and the abundant expression of high-affinity receptors for IgE [40,41]. In studies of food allergies, CML acts as an immunogen by inducing the activation and proliferation of various immune cells and participates in the development of chronic inflammation [42,43]. Our results show that IgE correlated positively with CML and that there existed a significant interaction of IgE and CML in relation to ISR, suggesting that elevated circulating CML might contribute to the activation of granular cells and the amplification of inflammation, mediating more local and systemic IgE release and, at the same time, leading to the pathogenesis of ISR in type 2 diabetic patients.

### 4.2. Potential Clinical Implications

The findings of the present study are of clinical relevance. Our results suggest that the measurement of IgE and CML is useful for evaluating the risk of ISR in patients with T2DM undergoing DES-based PCI. More importantly, aggressive glycemic control and anti-allergic and anti-inflammatory therapy may be mandatory to reduce ISR—especially for individuals with high levels of IgE or CML. Further prospective studies with large cohorts are warranted to confirm these issues.

### 4.3. Limitations

The present study has several limitations inherited from its retrospective, cross-sectional design formed for the purposes of ISR investigation—allowing us to detect associations, not to formulate causal links. The sample size in the ISR and non-ISR groups was small, and all patients were specially selected. Although baseline clinical characteristics and angiographic features were quite homogenously distributed in patients with and without ISR, certain selection biases and unknown confounding factors possibly impacting IgE and CML could not be excluded. ISR was determined by the interpretation of angiography and not through intracoronary imaging (such as intravascular ultrasound); thus, we could not provide details on the degree of ISR. Finally, the relationship between serum IgE and CML levels and ISR would be more precisely characterized by serial biochemical measurements.

## 5. Conclusions

This study demonstrates that in patients with T2DM, elevated serum IgE and CML levels confer an increased risk of ISR after DES-based PCI. This novel information provides new insights into the pathophysiology of ISR and the management of type 2 diabetic patients with stable coronary artery disease.

## Figures and Tables

**Table 1:** Baseline characteristics in patients with T2DM.

	Non–ISR (*n* = 220)	ISR (*n* = 196)	*p* Value
Male, *n* (%)	168 (76.4)	143 (73.0)	0.425
Age, y	66.53 ± 9.43	67.59 ± 8.82	0.241
Body mass index, kg/m^2^	25.50 ± 3.42	25.10 ± 3.46	0.235
Smoking, *n* (%)	63 (28.6)	59 (30.1)	0.743
Hypertension, *n* (%)	169 (76.8)	160 (81.6)	0.228
Systolic blood pressure, mm Hg	137.85 ± 20.40	140.26 ± 22.04	0.248
Diastolic blood pressure, mm Hg	74.41 ± 12.58	75.43 ± 14.69	0.448
Dyslipidemia, *n* (%)	25 (11.4)	20 (10.2)	0.704
Serum creatinine, µmol/L	80.00 (70.25–93.00)	82.50 (68.00–99.75)	0.518
eGFR, mL/min per 1.73 m^2^	80.00 ± 17.26	78.77 ± 33.05	0.640
HbA1c, %	7.33 ± 1.35	7.64 ± 1.35	0.020
Fasting blood glucose, mmol/L	7.15 ± 2.28	7.66 ± 2.94	0.050
Triglyceride, mmol/L	1.75 ± 1.72	1.64 ± 1.16	0.442
Total cholesterol, mmol/L	3.62 ± 1.01	3.59 ± 1.03	0.823
HDL cholesterol, mmol/L	1.09 ± 0.27	1.04 ± 0.26	0.054
LDL cholesterol, mmol/L	2.04 ± 0.80	2.02 ± 0.85	0.877
hsCRP, mg/L	0.94 (0.41–2.24)	1.15 (0.52–4.59)	0.021
IgE, ng/mL	80.25 (30.65–202.50)	187.10 (63.75–489.65)	<0.001
CML, ng/mL	174.26 (130.85–215.56)	203.26 (164.50–266.84)	<0.001
Ejection fraction, %	62.46 ± 8.89	60.34 ± 10.54	0.028
Medication, *n* (%)			
Dual antiplatelet therapy	209 (95.0)	185 (94.4)	0.781
ACEI/ARB	146 (66.4)	132 (67.3)	0.832
β-Blockers	183 (83.2)	159 (81.1)	0.584
Statins	217 (98.6)	184 (93.9)	0.009
Diseased vessel, *n* (%)			
Left main	11 (5.0)	19 (9.7)	0.065
Left anterior descending	157 (71.4)	153 (78.1)	0.118
Left circumflex	98 (44.5)	115 (58.7)	0.004
Right coronary artery	108 (49.1)	138 (70.4)	<0.001
Severity of CAD, *n* (%)			
1-vessel	114 (51.8)	56 (28.6)	<0.001
2-vessel	69 (31.4)	70 (35.7)	0.348
3-vessel	37 (16.8)	70 (35.7)	<0.001
Multivessel disease	106 (48.2)	140 (71.4)	<0.001
Lesion characteristics			
Class B2/C lesion, *n* (%)	135 (61.4)	145 (74.0)	0.006
Bifurcation lesion, *n* (%)	54 (24.5)	71 (36.2)	0.009
Chronic total occlusion, *n* (%)	23 (10.5)	36 (18.4)	0.021
Pre-PCI stenosis, %	85.86 ± 6.29	86.81 ± 8.20	0.189
Average number of stents, n	1.51 ± 0.71	1.63 ± 0.73	0.106
DES-zotarolimus, *n* (%)	98 (44.5)	86 (43.9)	0.891
DES-everolimus, *n* (%)	78 (35.5)	76 (38.8)	0.484
DES-sirolimus, *n* (%)	44 (20.0)	34 (17.3)	0.489
Stent diameter, mm	2.98 ± 0.40	2.84 ± 0.35	<0.001
Stent length, mm	28.52 ± 2.57	28.13 ± 4.94	0.322
Follow-up duration, months	12.12 ± 0.82	12.23 ± 0.88	0.161

T2DM: Type 2 diabetes mellitus; ISR: in-stent restenosis; eGFR: estimated glomerular filtration rate; HbA1c: glycated hemoglobin A1c; HDL: high-density lipoprotein; LDL: low-density lipoprotein; hsCRP: high-sensitivity C reactive protein; IgE: immunoglobulin E; CML: Nε-carboxymethyl-lysine; ACEI/ARB: angiotensin-converting enzyme inhibitor/angiotensin receptor blocker; PCI: percutaneous coronary intervention; DES: drug-eluting stent.

**Table 2:** Correlation between CML and IgE in patients with T2DM.

	Unadjusted r	Unadjusted *p*	* Adjusted r	* Adjusted *p*
All	0.331	<0.001	0.324	<0.001
ISR	0.433	<0.001	0.441	<0.001
Non-ISR	0.153	0.023	0.169	0.014

* Adjusted for age, sex, body mass index, smoking, dyslipidemia, hypertension, HbA1c, LVEF, statin use, number of diseased vessel, class B2/C lesion, bifurcation lesion, CTO lesion, and stent diameter. T2DM: type 2 diabetes mellitus; ISR: in-stent restenosis; HbA1c: glycated hemoglobin A1c; LVEF: left ventricular ejection fraction; CTO: chronic total occlusion.

**Table 3:** Uni- and multi-variant regression models.

	Unadjusted OR	*p*-Value	Adjusted for Model 1 OR	*p*-Value	Adjusted for Model 2 OR	*p*-Value
Log_2_ IgE per SD	2.008 (1.613–2.500)	<0.001	2.066 (1.652–2.583)	<0.001	1.989 (1.567–2.526)	<0.001
IgE tertiles	1.831 (1.431–2.344)	<0.001	1.879 (1.461–2.417)	<0.001	1.773 (1.355–2.322)	<0.001
1st	Ref		Ref		Ref	
2st	1.658 (1.019–2.697)	0.042	1.757 (1.070–2.884)	0.026	1.646 (0.971–2.790)	0.064
3st	3.346 (2.043–5.480)	<0.001	3.523 (2.129–5.829)	<0.001	3.137 (1.831–5.374)	<0.001
Log_2_ CML per SD	1.818 (1.445–2.287)	<0.001	1.824 (1.444–2.303)	<0.001	1.945 (1.507–2.509)	<0.001
CML tertiles	1.617 (1.268–2.061)	<0.001	1.608 (1.256–2.060)	<0.001	1.744 (1.333–2.282)	<0.001
1st	Ref		Ref		Ref	
2st	1.334 (0.824–2.159)	0.241	1.301 (0.797–2.124)	0.292	1.543 (0.902–2.641)	0.113
3st	2.605 (1.604–4.231)	<0.001	2.574 (1.570–4.221)	<0.001	3.026 (1.768–5.179)	<0.001

**Table 4:** The predictive power of models for ISR.

	C-Statistic	*p*-Value	*p* _for comparison_	Categorical NRI	*p*-Value	IDI	*p*-Value
Established risk factors	0.705 (0.655–0.755)	<0.001	Ref		Ref		Ref
Established risk factors + IgE	0.759 (0.713–0.804)	<0.001	<0.01	0.120 (0.047–0.194)	<0.01	0.074 (0.049–0.010)	<0.01
Established risk factors + CML	0.748 (0.701–0.794)	<0.001	<0.01	0.099 (0.034–0.163)	<0.01	0.062 (0.038–0.086)	<0.01

## Data Availability

Data generated or analyzed during this study are included in this published article.
